# Real-Time Tau Protein Detection by Sandwich-Based Piezoelectric Biosensing: Exploring Tubulin as a Mass Enhancer

**DOI:** 10.3390/s18040946

**Published:** 2018-03-22

**Authors:** Dujuan Li, Simona Scarano, Samuele Lisi, Pasquale Palladino, Maria Minunni

**Affiliations:** 1 College of Life Information Science & Instrument Engineering, Hangzhou Dianzi University, 115 Wenyi Rd, Hangzhou 310000, China; dujuanli2015@outlook.com; 2 Department of Chemistry “Ugo Schiff”, University of Florence, via della Lastruccia 3-13, Sesto Fiorentino, 50019 Firenze, Italy; samuele.lisi@outlook.com (S.L.); pasquale.palladino@unifi.it (P.P.); maria.minunni@unifi.it (M.M.)

**Keywords:** tau protein, Alzheimer’s disease, quartz crystal balance (QCM), immunosensor, tubulin, cerebrospinal fluid

## Abstract

Human tau protein is one of the most advanced and accepted biomarkers for AD and tauopathies diagnosis in general. In this work, a quartz crystal balance (QCM) immunosensor was developed for the detection of human tau protein in buffer and artificial cerebrospinal fluid (aCSF), through both direct and sandwich assays. Starting from a conventional immuno-based sandwich strategy, two monoclonal antibodies recognizing different epitopes of tau protein were used, achieving a detection limit for the direct assay in nanomolar range both in HBES-EP and aCSF. Afterward, for exploring alternative specific receptors as secondary recognition elements for tau protein biosensing, we tested tubulin and compared its behavior to a conventional secondary antibody in the sandwich assay. Tau–tubulin binding has shown an extended working range coupled to a signal improvement in comparison with the conventional secondary antibody-based approach, showing a dose–response trend at lower tau concentration than is usually investigated and closer to the physiological levels in the reference matrix for protein tau biomarker. Our results open up new and encouraging perspectives for the use of tubulin as an alternative receptor for tau protein with interesting features due to the possibility of taking advantage of its polymerization and reversible binding to this key hallmark of Alzheimer’s disease.

## 1. Introduction

**Figure 1:**
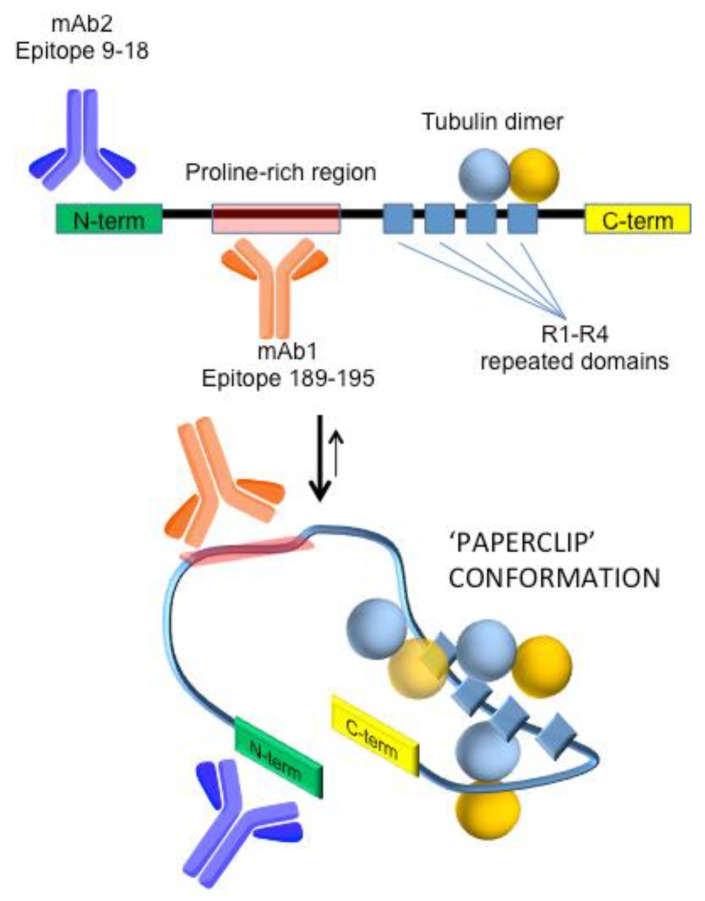
Sketched representation of tau protein’s domains, conformation in solution, and regions involved in the specific recognition by the bioreceptors used in this work. Namely, monoclonal antibody mAb1, which maps the proline-rich domain (residues 189–195), mAb2, which binds the *N*-terminal region (residues 9–18), and tubulin dimers that bind the R1–R4 repeated domain.

The World Alzheimer Report 2016 [1] estimates that dementia syndromes affect over 46 million people worldwide, and approximately 50% of them are diagnosed with Alzheimer’s disease (AD). AD-related pathophysiological changes can be detected as early as 10–20 years before the appearance of cognitive impairment. The detection of these changes by biochemical assays measuring levels of biomarkers in the cerebrospinal fluid (CSF), up to now the reference matrix for analysis of AD biomarkers, has led to a conceptual shift in the field of early Alzheimer’s disease diagnostics [2,3]. Human tau protein is one of the most advanced and accepted biomarkers for AD and tauopathies diagnosis in general [4,5,6,7], attracting increasing interest for developing anticipated and accurate molecular diagnostics [8,9,10,11]. Bioanalytical approaches to tau protein detection have recently been reviewed, and we refer readers to these works for a detailed description of the currently available analytical platforms [4,8]. Tau protein (50–65 kDa) belongs to the microtubule-associated protein family (MAP) and is expressed in human neurons and glia cells as oligodendrocytes and astrocytes. Tau protein interacts with tubulin, with a role in microtubules polymerization and stabilization. Its structure can be divided into four regions (Figure 1), the *N*-terminal region; the proline-rich domain; the microtubule-binding domain; and the C-terminal region, preferentially adopting a ‘paperclip’ conformation in solution [12].

At present, the available detection methods for tau protein are based in large part on immuno-based recognition. Declared ranges for calibrators of enzyme-linked immunosorbent assay (ELISA)-based kits are 50–2000 ng L^−1^ for total tau, with a limit of detection (LOD) = 34 ng L^−1^ for all six protein isoforms. Very recently, the diagnostic role of non-phosphorylated tau in CSF has been addressed and an LOD of 25 ng L^−1^ has been reported [2]. A nanoplatform used for Surface-Enhanced Raman Spectroscopy (SERS) fingerprint identification of β-amyloid and tau protein in blood with detection limit at 0.1 ng L^−1^ has also been described [13]. Moreover, two ultrasensitive assays have been reported [10,11]. The first is related to the detection of 3-repeat (3R) tau isoforms in Pick’s disease brain extracts and undiluted cerebrospinal fluid based on real-time fluorescence detection of thioflavin T (ThT) incorporated by protein seeds [10]. The second allows the direct and rapid fluorescence detection of β-amyloid, tau, and phosphorylated tau in CSF, saliva, serum, and urine using magnetic nanoparticles [11].

In the new diagnostic framework for preclinical Alzheimer’s disease, comprising biomarker-based research criteria developed by the workgroups of the National Institute on Aging–Alzheimer’s Association (NIA-AA) and those proposed by the International Working Group (IWG-2), the availability of label-free, real-time and low-cost methods based on biosensing tau biomarker in CSF represents an important possibility. Very recently, we reported Surface Plasmon Resonance (SPR)-based sensing of tau protein at the nanomolar level in artificial CSF (aCSF) by employing Multi-Walled Carbon Nanotubes (MWCNTs) in a sandwich-like detection strategy to enhance the analytical performances of the biosensor [14]. Although affinity sensing based on QCM transduction has been successfully applied to aggregation kinetic studies [15] and AD clinical diagnostics [16,17] based on amyloid-β detection, to our best knowledge its application to tau protein detection has not been reported so far. Contrarily, some encouraging findings with electrochemical [18,19,20] and conventional and/or localized SPR-based [14,21,22] have been reported in the recent past. QCM relies on a quartz crystal alternating current-induced oscillations with an inverse relationship between the oscillation frequency and interface phenomena such as mass loading on the surface or changes of the viscosity or density of the media surrounding the sensor. For this reason, biochemical interactions can be directly displayed in real time and without the use of any label, allowing for simple detection of key biomarkers using consolidated chemistries, e.g., thiol chemistry coupled to amino coupling for receptor attachment, and several assay formats. In this context, we explored for the first time QCM capabilities in tau detection through the development of an immunosensor for tau protein with application in a simulated matrix (aCSF). Direct and sandwich-based detection strategies are compared here by using two monoclonal antibodies able to recognize different epitopes, as represented in Figure 1. Furthermore, we exploited the well-known and specific recognition between tau and tubulin as an alternative to classical immuno-based sandwich assays. To the best of our knowledge, this is the first attempt at using tubulin as a recognition element by piezoelectric biosensing.

## 2. Materials and Methods 

### 2.1. Reagents and Buffers

Human tau protein (isoform 2N4R, MW 46 kDa) was purchased from Enzo Life Sciences (Lyon, France). Primary antibody (mAb1, monoclonal, clone 39E10 produced in mouse) was from Biolegend (San Diego, CA, USA). This antibody recognizes the middle domain (amino acids 189–195) shared by all isoforms of tau protein. Secondary monoclonal antibody (mAb2, monoclonal, clone Tau12, produced in mouse) was purchased from Merck (Daarmstaad, Germany) and recognizes the *N*-terminus of tau. Human serum albumin (HSA) was from Sigma-Aldrich, Milan, Italy. Tubulin (bovine brain, >99% pure, lyophilized) was from Cytoskeleton Inc. and purchased from Società Italiana Chimici, Rome, Italy. Tubulin consists of a heterodimer of one alpha and one beta isotype, each tubulin isotype is 55 kDa in size. Typically, the molar equivalent of tubulin is defined as the heterodimer that has a molecular weight of 110 kDa. 11-mercapto-1-undecanoic acid (MUA), 1-ethyl-3-(3-dimethyl-aminopropyl) carbodiimide (EDAC) and ethanolamine HCl (EA) used for the modification of the gold surface of the sensor and for the immobilization of the antibodies were purchased from Sigma (Milan, Italy) as well as *N*-2-hydroxyethylpiperazine-*N*′-2-ethanesulfonic acid (HEPES), 1,4-Piperazinediethanesulfonic acid (PIPES), 2-(*N*-morpholino)ethanesulfonic acid (MES) hydrate, Ethylene glycol-bis(2-aminoethylether)-*N*,*N*,*N*′,*N*′-tetraacetic acid (EGTA), Guanosine 5′-triphosphate sodium salt hydrate (GTP) and glycerol. *N*-hydroxysuccinimide (NHS) was from Fluka (Milan, Italy). Ammonia (28%) and hydrogen peroxide (30%) were obtained from Merck (Milan, Italy). Ethanol, sodium acetate and all the reagents for the buffers were purchased from Merck (Italy). All solutions were prepared using double-distilled MilliQ water, unless otherwise stated. Antibody immobilization buffer was 10 mM CH_3_COONa (pH 4.5). Artificial cerebrospinal fluid (aCSF) consisted in 150 mM NaCl, 3 mM KCl, 1.4 mM CaCl_2_, 0.8 mM MgCl_2_, 0.8 mM Na_2_HPO_4_, 0.2 mM NaH_2_PO_4_ with 100 mg L^−1^ HSA, pH 7.3. The HBS-EP buffer (pH 7.4) contained 10 mM HEPES-Na, 150 mM NaCl, 3 mM EDTA and 0.005% Tween 20. Binding tests with tubulin were carried out in different buffer solutions: Phosphate buffer solution (PB, 20 mM Na_2_HPO_4_, 20 mM NaH_2_PO_4_, pH 7.2); MES solution (200 mM MES, pH 6.5); PEM buffer (80 mM PIPES, 2 mM MgCl_2_ 0.5 mM EGTA, and 1 mM GTP, pH 6.9).

### 2.2. Piezoelectric Apparatus 

The quartz crystal analyzer used for the measurements was the Model QCA922 (Seiko EG&G, Chiba, Japan). Sensorgrams were recorded in real time by a computer connected to the instrument interface using home-made software.

### 2.3. Sensor Chip Functionalization and Detection Format

Quartz crystals (9.5 MHz AT-Cut, 14 mm) with gold evaporated (42.6 mm^2^ area) onto both sides were purchased from Elbatech (Marciana (LI), Italy). The gold surface of the sensor chip was modified through thiol self-assembled monolayer (SAM) to which the primary monoclonal antibody (mAb1) was attached. Prior to use, the quartz crystal was cleaned for 10 min in a boiling solution of H_2_O_2_ (30%), NH_3_ (28%) and MilliQ water in a 1:1:5 ratio; then thoroughly washed with distilled water, dried, and used immediately afterward. The crystal was then immersed in 1 mM MUA ethanol solution for 48 h at room temperature to allow film formation via the strong Au–thiol bond with the tail carboxylic group exposed at the monolayer–liquid interface. Then the crystal was rinsed thoroughly with ethanol and sonicated in ethanol for 10 min. After rinsing with ethanol and drying with nitrogen, the MUA modified crystal was fixed into a methacrylate cell, where only one side of the crystal was in contact with the solution. The immobilization chemistry involved the covalent peptide bond forming process. The activation of the carboxylic groups was achieved by reacting the MUA-modified surface with an aqueous solution of 50 mM NHS and 200 mM EDAC for 15 min. An aliquot (100 µL) of mAb1 (100 mg L^−1^) was introduced to the activated MUA surface and lasted for 40 min. The surface was then rinsed with buffer to remove nonspecifically bound mAb1 before adding 1 M EA (pH 8.5) for 15 min to block any remaining active esters on the surface. After baseline equilibration, the crystal was ready for measurements.

### 2.4. Detection of Tau Protein 

All measurements were carried out at room temperature (~20 °C) and in static conditions. Frequency shifts (in Hertz) due to the mass change at the sensing surface were measured by subtracting the baseline frequency recorded in the proper blank solution (HBS-EP buffer or simulated matrix, i.e., aCSF) from the frequency recorded after the binding of the relative ligands, i.e., tau protein, the secondary monoclonal Ab (mAb2), or tubulin. In the direct assay, tau protein (100 µL in HBS-EP buffer or aCSF) at different concentrations was added to the sensing surface and left in contact with the immobilized primary antibody for 15 min. The surface was washed to remove the excess of unbound protein and the change in frequency signals before and after protein incubation was recorded. For the sandwich-based assay, 100 µL of secondary antibody (mAb2, 10 mg L^−1^ in HBS-EP buffer) were added to the sensing surface after the binding of tau with the mAb1 and incubated for 15 min. Subsequently, the immunocomplex was washed to remove unbound mAb2. The frequency change was measured before and after each addition to evaluate the relative binding shifts. When the sandwich strategy was carried out, mAb2 was first used as a secondary binder. Alternatively, tubulin was explored as a secondary receptor (100 µL of 5 µM tubulin solution in PB, MES, or PEM buffers) and added to the sensing surface after the formation of the tau–mAb1 complex. After incubation (15 min), the surface was rinsed with buffer. In all the measurements, the surface of the biosensor was regenerated with 20 mM NaOH and 20 mM HCl in sequence for 30 s and equilibrated in buffer before starting a new measuring cycle.

## 3. Results and Discussion

With the aim to develop an immuno-based biosensor by QCM transduction, we selected two monoclonal antibodies able to bind tau protein on different epitopes (Figure 1). The primary mAb (mAb1) recognizes tau protein in its middle domain (amino acids 189–195) and was used as a capturing receptor for its direct detection by QCM measurements (Figure 1). Once bound to the analyte, it leaves the target protein free for further binding by means of secondary recognition to improve the selectivity and limit of detection of the bioassay. This step was achieved by using a secondary mAb (mAb2). The analytical performances of the direct detection (via mAb1 recognition) and the sandwich-based assay (mAb1/Tau/mAb2 recognition) were directly compared. Finally, an original modification of the classical sandwich strategy was performed by substituting the mAb2 with tubulin, as an alternative natural recognition element.

### 3.1. Direct Immuno-Based Detection of Tau in Buffer and aCSF

**Figure 2:**
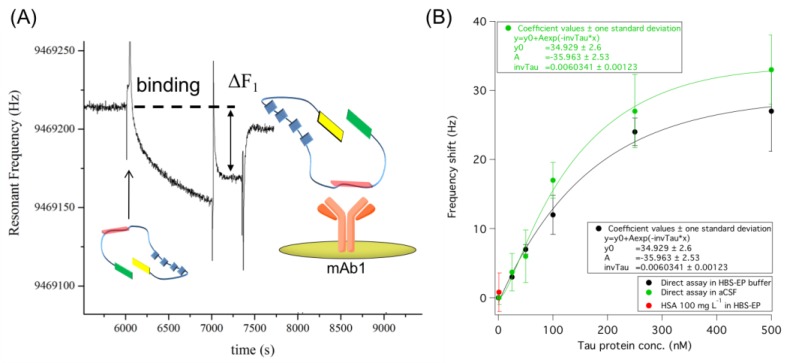
(**A**) Raw sensorgram relative to the direct assay of tau protein on the piezoelectric biosensor carrying mAb1 as a primary receptor. Tau addition on the sensor and the subsequent washing with buffer are underlined. (**B**) Dose–response trend of the immunosensor with the relative exponential trend obtained within 0–500 nM tau concentration range in HBS-EP buffer (black) and in aCSF (green), and HBS-EP buffer spiked with 100 mg L^−1^ HSA as negative control (red). SDs are calculated from *n* = 5 independent measurements.

We have compared the tau binding capability of the HBS-EP buffer or aCSF, as previously reported, by using SPR transduction under the same conditions [14]. Compared with conventional binding buffers, both the stability and the binding ability of this protein were improved in aCSF. Therefore, we first compared the dose–response relationship of tau in HBS-EP buffer and aCSF in the range 0–500 nM, as reported in Figure 2B.

The obtained data displayed very similar trends of tau binding in the two tested solutions, with a slightly better performance from the aCSF matrix, both in terms of frequency shifts and reproducibility. These results are in agreement with those previously reported by SPR analysis [14]. The whole dynamic range of the biosensor can be described by an exponential trend (Figure 2B). However, for the low concentration range of interest in real sample analysis (0–250 nM), the behavior can be described well by linear fitting both in HBS-EP and aCSF (Figure S1). The detection limits estimated on the basis of the linear equations in Figure S1 were 50 nM (y = 1.20 + 0.09x) and 42 nM (y = 1.53 + 0.11x) for HBS-EP and aCSF, respectively. On the whole, with respect to the same approach by SPR detection [14], the piezoelectric-based assay displayed a wider linear response (SPR: 125 nM and 30 nM for HBS-EP and aCSF, respectively) but a lower sensitivity (SPR: about 8 nM and 15 nM for HBS-EP and aCSF, respectively). No matrix effect was observed when aCSF was tested alone (Figure 2B). Considering that at present cerebrospinal fluid (CSF) is the only matrix validated for tau protein detection, we also tested the biosensor behavior in the presence of Human Serum Albumin (HSA), i.e., the most representative protein in CSF. Spiked HBS-EP buffer solutions (100 mg L^−1^) tested as negative controls gave a negligible signal and confirmed the selectivity of mAb1 for tau protein (the red dot in Figure 2B).

### 3.2. Improving Sensor Performances by Affinity-Based Mass Enhancement 

A secondary ligand, binding tau in a different epitope than the primary antibody, was used in a sandwich-like assay applying two different mass enhancers: a secondary monoclonal antibody (mAb2), as already assessed by our group by using SPR detection [14], or the tubulin protein, previously employed as pre-polymerized microtubules (MT), to understand neurological disease development processes [23].

#### 3.2.1. Secondary Monoclonal Antibody

**Figure 3:**
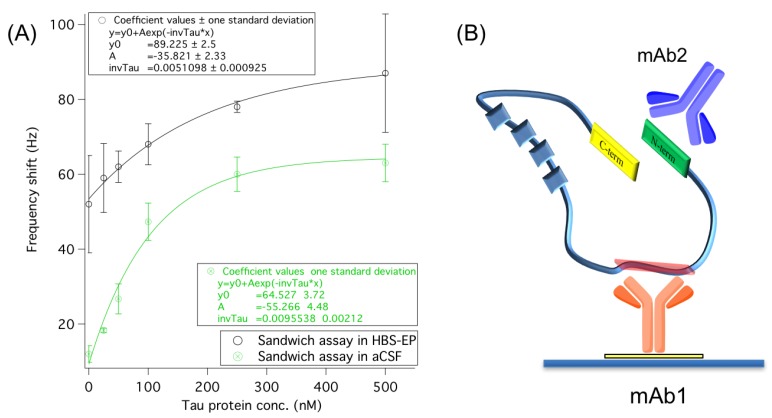
(**A**) Dose–response trends obtained by the immuno-based sandwich assay (with mAb2 as secondary receptor) of tau protein performed in HBS-EP buffer or aCSF matrix on the piezoelectric biosensor. Data refer to the final frequency shifts obtained by the sandwich approach. SDs are calculated from *n* = 3 independent measurements. (**B**) Representation of the immuno-based sandwich assay of tau. The primary antibody (mAb1) attached on sensor chip binds the middle domain (amino acids 189–195) of tau protein highlighted in red. Subsequently, the secondary antibody (mAb2) binds the *N*-terminus of tau protein depicted in green.

Following the classical approach, a secondary monoclonal antibody (mAb2) recognizing the *N*-terminus region of tau protein was used to perform the sandwich format assay. Tau protein was tested in both HBS-EP buffer and aCSF matrix. After tau interaction with the primary antibody (mAb1), the secondary antibody was added to the measurement cell for 15 min. Afterward, the excess was removed and washed with buffer, and the final frequency shifts were recorded. In Figure 3A the comparison of the direct and sandwich-based detection is reported in the two conditions, i.e., HBS-EP or aCSF. Accordingly to the dose–response relationship observed, and in agreement with our previous results [14], it can be seen that HBS-EP buffer significantly affects the reproducibility and the specificity of the dose–response behavior. This could be attributed to a lower stability of tau protein in HBS-EP during measurements, which may contribute to undesired conformation changes or aggregation phenomena of tau in solution. This, in turn may impact on unspecific analyte adsorption on the surface, as suggested by the high, significant negative control signal (i.e., mAb2 tested directly on mAb1 in the absence of tau protein) of 52 ± 13 Hz (*n* = 3). The high variability over measurements supports this hypothesis. Contrarily, in the aCSF matrix the enhanced signals show a dynamic dose–response relationship up to 250 nM (Figure 3A), and an estimated linear range up to 100 nM (see Figure S2). Standard deviations recorded in aCSF are significantly lower than those recorded in HBS-EP, and harmonic over the investigated concentration range. This clearly indicates the selective nature of the secondary binding. The dose–response relationship in aCSF can be successfully fitted by an exponential trend over the whole concentration range, by which the improved limit of detection was estimated down to about 10 nM for tau protein.

#### 3.2.2. Use of Tubulin as an Alternative to Antibodies in a Sandwich-Like Assay 

The rational of using tubulin as a secondary binder for tau protein is based on the favorable stoichiometry and the high specificity and affinity of this recognition [24,25,26]. Ackmann et al. [24] reported for this interaction a K_D_ = 75 ± 30 nM with a stoichiometry (defined by factor *n* = [tau]bound/[MT]) of 0.20 for tau protein in 5–500 nM range, which is exactly where we focused on detecting tau in aCSF. Moreover, the possibility of controlling the in situ tubulin polymerization and the reversibility of the process would give good opportunities to control the system in terms of sensor reuse. The tau–tubulin binding (mAb1 as primary receptor) was tested in different buffers to find effective operating conditions. First the PB buffer, widely used for biomolecular interactions, was adopted for dissolving tubulin. Surprisingly, no secondary signal due to tubulin dimers binding to tau was observed (5 ± 2 Hz, *n* = 3). The same result was obtained in aCSF. Therefore, we tested solutions employed in studies on tubulin that showed that Mg^2+^ salts [27] and GTP (guanosine 5′-triphosphate) in PIPES [28], PEM [29] and MES [30] buffers efficiently trigger tubulin polymerization. We found important unspecific binding in MES buffer when aCSF was spiked with 100 mg L^−1^ HSA, corresponding to 42 ± 5 Hz (*n* = 3), likely due to an unspecific interaction between tubulin and HAS in solution. On the contrary, negligible interference (2 ± 1 Hz, *n* = 3) was observed in PEM. For this reason, the PEM buffer was selected for subsequent tau–tubulin interaction measurements.

**Figure 4:**
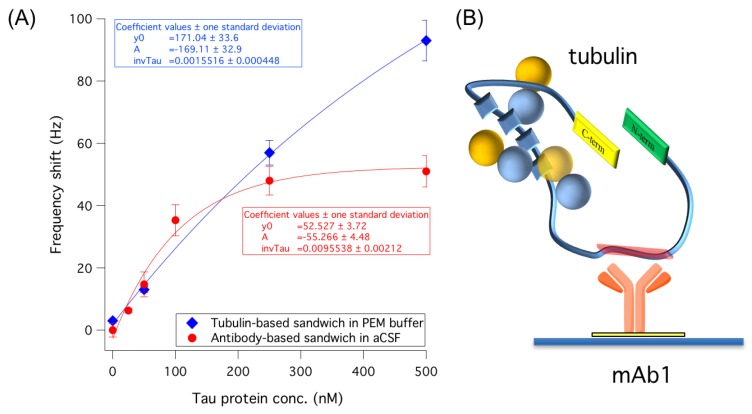
(**A**) Dose–response trends with the relative exponential fitting curves obtained by sandwich assay (0–500 nM tau) by using tubulin in PEM buffer (blue) or mAb2 in aCSF (red) for the signal enhancement on the piezoelectric biosensor. Unspecific signals were subtracted. SDs are calculated from *n* = 3 independent measurements. (**B**) Representation of the assay, in which the primary antibody (mAb1) attached on the sensor chip binds the middle domain (amino acids 189–195) of tau protein. Subsequently, tubulin dimers (yellow and blue spheres) bind the R1–R4 region (blue squares) of tau protein.

In consideration of the well-known possible rearrangement of tau protein in the so-called ‘paperclip’ conformation, we also evaluated the possibility that tubulin binding could be impaired by the non-favorable orientation of tau after its binding to the primary receptor. In particular, we hypothesized that the proper orientation of the protein after its binding to the primary receptor could be a key factor in the success of tubulin recognition. In fact, tubulin binding sites R1–R4 (Figure 1) could be oriented toward the sensor surface or exposed to the bulk environment depending on the mAb epitope involved in the primary binding. Therefore, we verified that our detection strategy involving the immobilization of mAb1 on the QCM surface was the most suitable for our purposes. To this end, in parallel experiments we immobilized mAb1 or mAb2 on different QCM surfaces to verify the best configuration of the assay. In this hypothesis, the use of mAb1 as a primary receptor should ensure the best exposure of the R1–R4 region to the bulk solution and therefore to tubulin (Figure S1). Obtained results conducted on 500 nM tau confirmed this assumption, showing negligible binding of tubulin when mAb2 was used as a primary receptor (data not shown). On the contrary, when mAb1 is used the tau–tubulin recognition is successfully achieved. The enhancement effect of tubulin binding was assayed on different tau concentrations after direct detection with mAb1, within 0–500 nM, giving the dose–response trend reported in Figure 4. As can be observed, at concentrations <150 nM the efficacy of tubulin or mAb2 used for signal enhancement is comparable, with similar detection limits (4.55 ± 0.45 Hz vs. 2.32 ± 1.83 Hz, 3s = 9). However, for higher concentrations of tau protein, a significant enhancement of the final signal (~two folds) is obtained with tubulin (blue spot) with respect to the classical immuno-based approach with mAb2 (red spot), together with an extended dynamic range. This effect could be due to the cooperative binding of tubulin dimers when tested in a solution buffer able to trigger their partial polymerization. The absence of unspecific interference due to the presence of HSA in aCSF matrix was assessed (on 500 nM tau protein), giving negligible binding (6 ± 5 Hz). On the whole, tubulin allows the selective targeting of tau protein and may be further explored for affinity-based approaches as a valid alternative to classic antibodies. The limited reproducibility observed here should be improved, e.g., working in flow to facilitate tau–tubulin complex removal from the surface. However, notably, the repeatability improves with a decrease in tau concentration, which could mean that the majority of the difficulty in regenerating the biosensor surface may be due to the concentration range investigated here.

## 4. Conclusions

QCM-based sensing for human tau protein in artificial cerebrospinal fluid (aCSF) has been achieved with a detection limit for the direct assay of 50 nM and 42 nM in HBES-EP and aCSF, respectively. The analytical performances of direct, classic sandwich, and tubulin-based secondary recognition strategies are tested and compared here. tau–tubulin binding has shown an extended working range coupled with signal improvement in comparison with the conventional secondary antibody-based approach. Tau–tubulin interaction conditions (PB, PIPES, MES, and PEM buffers; the presence of Mg^2+^ and GTP) were tested, with the best results in terms of non-specific adsorption and sensitivity recorded for the PEM buffer. Under the optimized conditions, tau within a 0–500 nM concentration range was assayed in aCSF, clearly showing a dose–response trend at a lower tau concentration than usually investigated (micromolar range) and closer to physiological levels in CSF, the reference matrix for protein tau biomarker, with important reflections on the real sensor applicability. In fact, the early identification of AD patients and the proper monitoring of the disease has a positive impact on defining effective therapeutic treatments and supplying patients with the most suitable treatment at the right time and dose. Our preliminary findings represent an interesting and innovative attempt to detecting Tau protein through a label-free, real-time, and low-cost biosensing strategy in a real matrix.

## Supplementary Materials

The following are available online at http://www.mdpi.com/1424-8220/18/4/946/s1, Figure S1: linear fitting of direct assay performed in HBS-EP buffer and aCSF; Figure S2: linear fitting of sandwich assay performed in aCSF; Figure S3: Sketched representation of the putative orientation of Tau protein after its primary recognition by antibody immobilized on the QCM surface.
