# Erratum: Li, D.; Scarano, S.; Lisi, S.; Palladino, P.; Minunni, M. Real-Time Tau Protein Detection by Sandwich-Based Piezoelectric Biosensing: Exploring Tubulin as a Mass Enhancer. *Sensors* 2018, *18*, 946

**DOI:** 10.3390/s19061428

**Published:** 2019-03-22

**Authors:** Dujuan Li, Simona Scarano, Samuele Lisi, Pasquale Palladino, Maria Minunni

**Affiliations:** 1 College of Life Information Science & Instrument Engineering, Hangzhou Dianzi University, 115 Wenyi Rd, Hangzhou 310000, China; dujuanli2015@outlook.com; 2 Department of Chemistry “Ugo Schiff”, University of Florence, via della Lastruccia 3–13, Sesto Fiorentino, 50019 Firenze, Italy; samuele.lisi@outlook.com (S.L.); pasquale.palladino@unifi.it (P.P.); maria.minunni@unifi.it (M.M.)

The authors wish to make the following erratum to this paper [1]:

There is one mistake in this article [1]. On page 9, there is correction needed in reference [14]:

“Lisi, S.; Scarano, S.; Fedeli, S.; Cicchi, S.; Pascale, E.; Ravelet, C.; Peyrin, E.; Minunni, M. Toward sensitive immuno-based detection of tau protein by surface plasmon resonance coupled to carbon nanostructures as signal amplifiers. *Biosens. Bioelectron.* **2016**, *85*, 83–89.”

This should be replaced with:

“Lisi, S., Scarano, S., Fedeli, S., Pascale, E., Cicchi, S., Ravelet, C., Peyrin, E.; Minunni, M. Toward sensitive immuno-based detection of tau protein by surface plasmon resonance coupled to carbon nanostructures as signal amplifiers. *Biosens. Bioelectron.* **2017**, *93*, 289–292.”

The authors would like to apologize for any inconvenience caused to the readers by these changes.
