# Supplementary material for: Real-Time Tau Protein Detection by Sandwich-Based Piezoelectric Biosensing: Exploring Tubulin as a Mass Enhancer
Source: Sensors (Basel). 2018 Mar 22;18(4):946. doi: 10.3390/s18040946 (PMC5948773; doi:10.3390/s18040946)
Supplement: Supplementary file 1 [file sensors-18-00946-s001.pdf]

Supplementary Materials

Real-Time Tau Protein Detection by Sandwich-Based Piezoelectric Biosensing: Exploring Tubulin as a Mass Enhancer

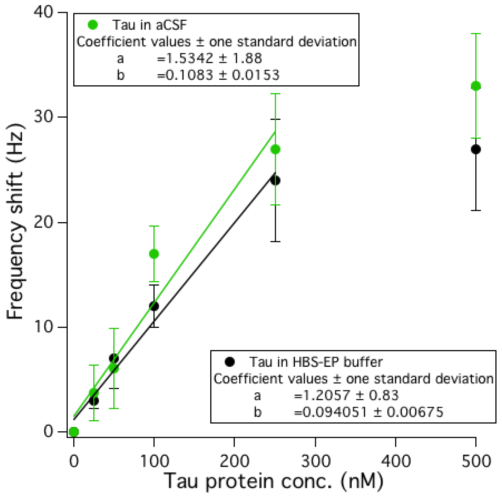

Figure S1. Linear correlation of the direct assay in HBS-EP buffer (black) and aCSF (green) calculated within the 0–250 nM range of tau concentration.

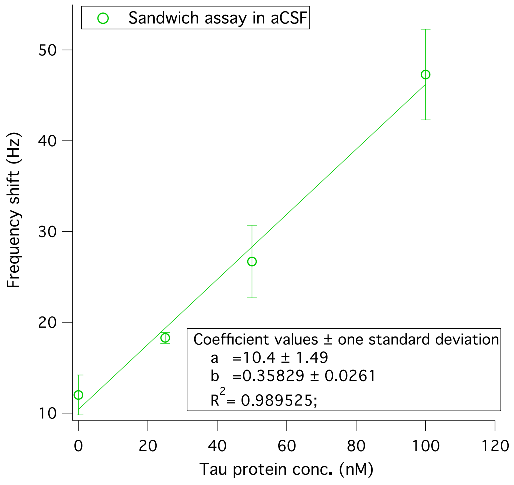

Figure S2. Linear correlation of the sandwich assay in aCSF calculated within the range 1–100 nM of Tau protein.

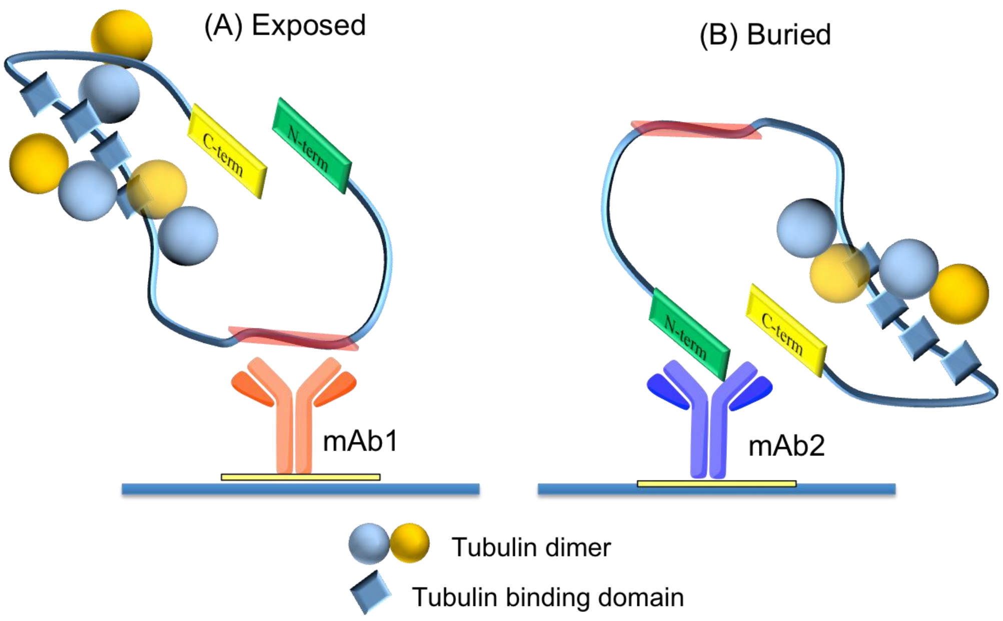

Figure S3. Sketched representation of the putative orientation of Tau protein after its primary recognition by antibody immobilized on the QCM surface. If mAb1 is used as the primary receptor (**A**), the repeated tubulin binding domain is exposed toward the bulk. On the contrary, the direct recognition of Tau by mAb2 leads to the unfavorable orientation of tubulin binding domain (**B**), which is likely exposed on the sensor surface. The two assets result in the ability or inability of tubulin to act as a secondary receptor in a sandwich format.
